# Automatic liver tumor segmentation on computed tomography for patient treatment planning and monitoring

**DOI:** 10.17179/excli2016-402

**Published:** 2016-06-27

**Authors:** Mehrdad Moghbel, Syamsiah Mashohor, Rozi Mahmud, M. Iqbal Bin Saripan

**Affiliations:** 1 Dept. of Computer & Communication Systems, Faculty of Engineering, University Putra Malaysia, 43400 Serdang, Selangor, Malaysia; 2 Cancer Resource & Education Center, University Putra Malaysia, 43400 Serdang, Selangor, Malaysia

**Keywords:** image segmentation, random walker segmentation, CT imaging, liver tumor burden, fuzzy c-means, cuckoo search optimization

## Abstract

Segmentation of liver tumors from Computed Tomography (CT) and tumor burden analysis play an important role in the choice of therapeutic strategies for liver diseases and treatment monitoring. In this paper, a new segmentation method for liver tumors from contrast-enhanced CT imaging is proposed. As manual segmentation of tumors for liver treatment planning is both labor intensive and time-consuming, a highly accurate automatic tumor segmentation is desired. The proposed framework is fully automatic requiring no user interaction. The proposed segmentation evaluated on real-world clinical data from patients is based on a hybrid method integrating cuckoo optimization and fuzzy c-means algorithm with random walkers algorithm. The accuracy of the proposed method was validated using a clinical liver dataset containing one of the highest numbers of tumors utilized for liver tumor segmentation containing 127 tumors in total with further validation of the results by a consultant radiologist. The proposed method was able to achieve one of the highest accuracies reported in the literature for liver tumor segmentation compared to other segmentation methods with a mean overlap error of 22.78 % and dice similarity coefficient of 0.75 in 3Dircadb dataset and a mean overlap error of 15.61 % and dice similarity coefficient of 0.81 in MIDAS dataset. The proposed method was able to outperform most other tumor segmentation methods reported in the literature while representing an overlap error improvement of 6 % compared to one of the best performing automatic methods in the literature. The proposed framework was able to provide consistently accurate results considering the number of tumors and the variations in tumor contrast enhancements and tumor appearances while the tumor burden was estimated with a mean error of 0.84 % in 3Dircadb dataset.

## Introduction

In many liver related clinical applications such as computer aided surgery and treatment planning, segmentation of liver and liver tumors is required. The need for a proper segmentation is further emphasized by the fact that liver cancer is amongst top cancers with the most fatalities (Grendell et al., 1996[30]; Habib et al., 2003[31]). Currently, contrast-enhanced computed tomography (CECT) is the most commonly used modality for liver imaging and monitoring (Hann et al., 2000[33]). However, manual identification and segmentation of all the tumors inside the liver is a tedious task resulting in segmentation and measurement of a subset of tumors to determine their size and location. In order to standardize this procedure, the Response Evaluation Criteria in Solid Tumors (RECIST) guideline has been proposed (Eisenhauer et al., 2009[24]). Based on the RECIST standard, only tumors where the largest axis is over 10 mm are considered for measurement with a decrease of 30 % or more in the axis length considered as a partial response to the treatment and an increase of 20 % or more considered as a progressive tumor. The main problem with RECIST standard as mentioned by Moltz et al. (2009[54]) and Bornemann et al. (2007[13]) is the fact that a single measurement of a diameter cannot correspond to the size of a 3D object such as a tumor, as these tumors are mostly asymmetrical in shape. In practice, a tumor size can be increased significantly if measured in 3D but its largest axis may not show a significant change in size. Some livers can have more than five tumors inside while based on RECIST standard, only five tumors are measured as an overall indication of the liver condition.

Unfortunately, due to the difficulties related to manual tumor segmentation and a compelling lack of publicly accessible datasets. Liver tumor segmentation methods have received less attention from researchers while many accurate liver segmentation methods have been proposed by many researchers (Heimann et al., 2009[35]; Linguraru et al., 2009[47]; Wang et al., 2015[72]; Anter et al., 2014[7]; Mostafa et al., 2015[55]; Shi et al., 2016[65]; Xu et al., 2015[74]). On the other hand, as manual segmentation of tumors by a radiologist is tedious and time-consuming, many published methods utilize datasets containing less than 20 segmented tumors. To increase interest in liver tumor segmentation, a challenge was presented by the Medical Image Computing and Computer Assisted Intervention Society (MICCAI) to segment liver tumors from clinical CT images. Liver Tumor Segmentation Challenge 2008 (LTSC '08) was a competition aimed at the development of several liver tumor segmentation algorithms. As a result of this competition, many automatic segmentation approaches were proposed, surpassing the accuracy of many semi-automatic methods published previously. However, the achieved accuracies can be considered lower than expected compared to the accuracy of liver segmentation methods (Heimann et al., 2009[35]). 

As a result of the LTSC'08, five semi-automatic, four automatic and one interactive segmentations were developed, as expected the interactive method based on a combination of graph-cuts and watershed algorithms proved to be the most accurate (Stawiaski et al., 2008[68]). Automatic methods include approaches based on machine learning and classification of voxels by the way of cognition networks (Schmidt et al., 2008[64]) and a method based on AdaBoost (Shimizu et al., 2008[66]). Semi-automatic methods included adaptive thresholding and morphological operations (Moltz et al., 2008[53]), propagational learning and voxel classification (Zhou et al., 2008[78]) and level-set segmentation (Smeets et al., 2008[67]). 

Recent publications include interactive tumor segmentation based on intensity distribution combined with hidden Markov fields (Häme and Pollari, 2012[32]), semi-automatic tumor segmentation with support vector machines with affinity constraint propagation (Freiman et al., 2011[26]) and machine learning algorithms (Xu and Suzuki, 2011[73]). Automatic segmentation includes a method utilizing region growing and watersheds (Anter et al., 2013[6]) and a method based on free-form deformations (Huang et al., 2014[38]). 

The LTSC '08 included a CECT dataset containing 20 tumors in total, unfortunately due to lack of maintenance on the LTSC'08 challenge website the dataset has become unavailable. As mentioned before, the lack of a common dataset resulted in publications on the subject being mostly from 2008 to 2010 with some publications afterward while their overall accuracy can be considered lower than desired. Recently, two datasets - one containing 120 tumors (Ircad, 2016[41]) and another one containing 10 tumors (Midas, 2016[51]) all with segmentations from expert radiologists - has been made publicly accessible for researchers. Hopefully, availability of these new datasets will motivate more researchers to focus on developing liver tumor segmentation methods, increasing the treatment prognosis of patients as accurate liver tumor and tumor burden estimation is an important part of liver treatment planning (Hopper et al., 2000[37]).

### Medical value of tumor segmentation

Percentage of tumor tissue present in the organ (in this case liver) called tumor burden is commonly utilized in both monitoring and assessment of pathological livers and can be used in the development of treatment strategies (Jagannath et al., 1986[42]). The tumor burden is also important as it can be used as an accurate representation of the effectiveness of different treatment protocols, increasing the treatment effectiveness (Gobbi et al., 2004[27]). 

Tumor burden is also utilized for evaluation of the effectiveness of cytotoxic anti-cancer drugs by radiologists (Prasad et al., 2002[61]) and in surgical intervention planning such as liver resection (Hopper et al., 2000[37]). As mentioned before, in clinical practice the tumor size is mostly calculated by measuring the maximum tumor axis in the transverse plane of CT image (Eisenhauer et al., 2009[24]). Let us assume the shape of the tumor as spherical, an increase of 20 % in the diameter will result in 72.8 % increase in volume while a decrease of 30 % in the tumor diameter will lead to 65.7 % decrease in tumor volume. In reality majority of tumors are shaped irregularly and while their largest diameter hardly changes, they can increase and decrease in size irregularly (Heckel et al., 2014[34]). As confirmed by various studies, it can be assumed that real 3D volume of a tumor is far more accurate for treatment planning (Bauknecht et al., 2010[9]; Bolte et al., 2007[12]; Bornemann et al., 2007[13]; Fabel et al., 2011[25]; Heussel et al., 2007[36]; Kuhnigk et al., 2006[45]; Puesken et al., 2010[62]) but due to difficulties associated with measuring it is not included in RECIST guidelines used by many radiologists. Most treatment planning is still based on manual or interactive segmentation of tumors based on RECIST guidelines with considerable inter-observer variations between different radiologists. This is apparent from the MIDAS dataset where there is almost 10 % disagreement in tumor borders between five professional radiologists.

Furthermore, a main constraint for the surgical resection planning is the lesion/liver ratio after surgical resection (Nordlinger et al., 1996[59]). Segmentation of the liver and tumors inside allow easier computation of this ratio, simplifying the planning for surgical resection. The identification of the regions to be removed becomes easier as tumors are well defined, the segmentation also provides a precise location of the tumors inside the anatomical segments of the liver simplifying the preoperational planning. In addition, liver and tumor segmentation also offers several applications for treatment planning, such as Thermal Percutaneous Ablation (Rossi et al., 1996[63]), Percutaneous Ethanol Injection (PEI) (Livraghi et al., 1995[48]), Radiotherapy Surgical Resection (Albain et al., 2009[2]) and Arterial Embolization (Yamada et al., 1983[75]). Furthermore, in treatments such as Selective Internal Radiation Therapy (SIRT) (Al-Nahhas et al., 2006[3]), fractional dose calculation of the liver and tumors depend on the volume of the liver and tumors. Hence in order to calculate the dose delivered to the tumor, it is essential to segment the tumor from the background and calculate the volume of the tumor region.

### Proposed method

The main advantage of an automatic segmentation over other segmentation methods is the reproducibility as no human interaction is required and the segmentation can run in the background without the need for any interaction from the user. An accurate Computer-aided detection/diagnosis (CAD) system with accurate segmentation methods for liver and liver tumors can have a great impact in the overall treatment planning of the patient as precise tumor volume and location estimation for all tumors inside the liver can result in the determination of the best course of treatment early on. 

In this paper, an automatic liver tumor segmentation is proposed based on contrast-enhanced computed tomography imaging. The proposed method based on a hybrid of fuzzy c-means algorithm with cuckoo optimization (CS-FCM) and random walkers method (RW) with priors was shown to have promising performance. The proposed segmentation is validated on publicly available clinical datasets with varying contrast and enhancements and further evaluated by a consultant radiologist to assess the clinical value of the proposed method. The performance of the proposed method and the low error in tumor burden determination compared to manual segmentation makes the proposed segmentation method a viable alternative to other segmentation methods. 

## Materials and Methods

### Dataset

**Figure 1:**
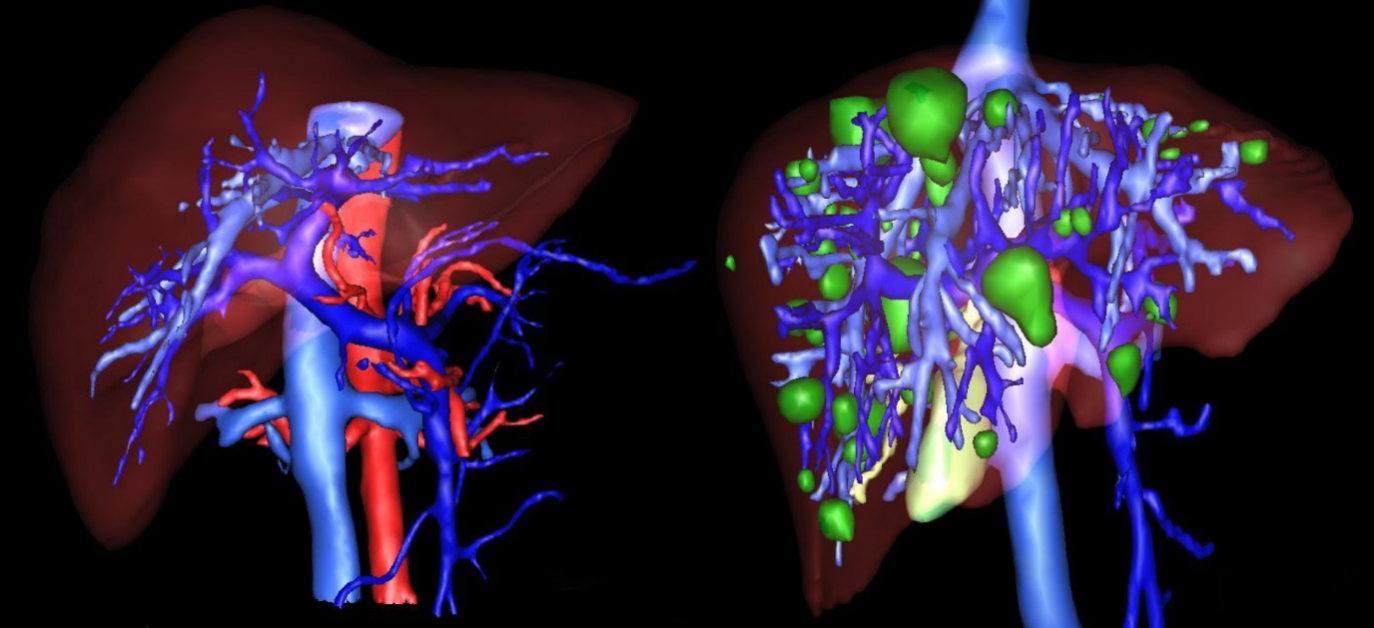
Healthy (left) versus pathological liver (right) from IRCAD dataset (tumors represented in green)

The proposed method is evaluated using 3Dircadb dataset from Research Institute against Digestive Cancer (IRCAD) (Ircad, 2016[41]) and The MIDAS liver tumor dataset from National Library of Medicine's Imaging Methods Assessment and Reporting (IMAR) project (Midas, 2016[51]). Figure 1(Fig. 1) illustrates a pathological liver from 3Dircadb dataset. All datasets used in tumor segmentation are acquired at different enhancement phases with various scanners.

Expert radiologists have manually outlined liver tumor contours for all images on a slice-by-slice basis in order to determine the ground truth. The number of slices in each series, the slice thickness and the pixel spacing varied from 64 to 502, 0.5 to 5.0 mm and 0.54 to 0.87 mm respectively with the image resolution being 512 × 512 in all cases. The 3Dircadb dataset is segmented by a single radiologist while the MIDAS dataset has the segmentation from five different radiologists; radiologist 1 was utilized as the ground truth in this study. However, 3 tumors from the 3Dircadb dataset are excluded from the segmentation as the tumor enhancement and contrast is not sufficient for automatic segmentation. All internal structures of the liver such as vessels and tumors are included in the liver mask during manual segmentations as the tumor segmentation is done inside the liver mask. A vessel is considered as a part of the liver if it is completely surrounded by the liver tissue. If a vessel is partially enclosed by the liver (often the case where large veins such as vena cava and portal vein enter or exit the liver), only the parts surrounded by liver tissue are included in the manual segmentation

**Figure 2:**
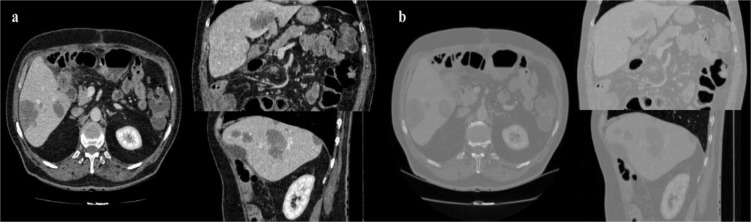
CT image with window level and settings recommendations for Abdominal CT (a), CT image with dynamic window level and settings (b)

It should be noted that the developed framework was run with Matlab 2013a on a personal computer with 8 GB of ram and an Intel i7 CPU. All the images utilized in this study are processed with window level recommendations for Abdominal CT imaging, as illustrated in Figure 2(Fig. 2).

### Image preprocessing

**Figure 3:**
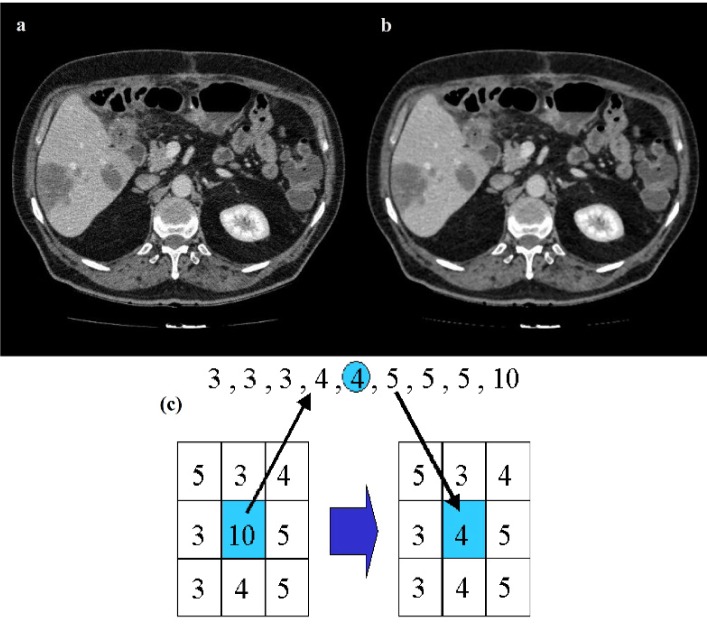
Results of median filtering, original CT image (a), filtered CT image (b), median filter (c)

A 3×3 Median filter is utilized for smoothing the images as shown in Figure 3(Fig. 3). The main reason of using the Median filtering for the preprocessing step of this algorithm is because Median filtering retains the edge information within the image where Mean filters and Gaussian filters tend to blur the edges in the image. This is because the Median filter does not create new unrealistic pixel values in the case of the filtering window laying over an edge. 

### Fuzzy clustering

Fuzzy set theorem, introduced by (Zadeh, 1965[77]) and its adaptation for image segmentation (Bezdek, 2013[11]) is amongst the most used and researched image segmentation algorithms.

In this paper, soft clustering (fuzzy c-means) is used as each pixel can belong to many clusters based on a membership degree resulting in better performance in images with poor contrast, region overlapping and inhomogeneity of region contrasts such as CT images, compared to hard clustering where each pixel can only belong to a single cluster. As traditional fuzzy c-means (FCM) algorithm where clustering is only based on pixel intensities is very sensitive to noise, addition of spatial relations between pixels has been proposed by many researchers to improve the performance (Ahmed et al., 2002[1]; Chen and Zhang, 2004[17]; Szilagyi et al., 2003[69]; Kang and Zhang, 2009[43]; Cai et al., 2007[15]; Krinidis and Chatzis, 2010[44]). 

The main disadvantage of FCM algorithm is its tendency to get trapped in local minima as it is very sensitive to initial solution (initial random cluster centers), metaheuristic approaches such as genetic algorithms (GA), tabu search (TS), simulated annealing (SA), ant colony based optimization (ACO) and their hybrids have been proposed by many researchers to overcome this limitation (Maulik and Bandyopadhyay, 2000[50]; Ng and Wong, 2002[56]; Niknam and Amiri, 2010[57]; Niknam et al., 2009[58]; Das et al., 2009[21]; Moh'd Alia et al., 2011[52]; Al-Sultan and Fedjki, 1997[4]; Benaichouche et al., 2013[10]). Metaheuristic optimization methods can perform well on noisy images and the initial solutions from them are often very close to optimal cluster centers (Dréo et al., 2006[23]). In this paper, a metaheuristic approach based on cuckoo search algorithm is proposed to increase the accuracy of liver tumor segmentation.

### Fuzzy c-means algorithm

As a fuzzy clustering method, fuzzy c-means algorithm is based on the representation of clusters by their respective centers. The data space *X* = {*x*_1_, *x*_2_, …, *x_N_*} can be clustered by minimizing the objective function *J* with respect to cluster centers and membership matrix *u* by:


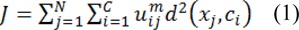


Based on following constraints:


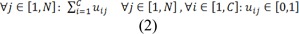


Where *U* = [*u_ij_*]*_C_*_×_*_N_* represents the membership function matrix, the distance between *x_j_* and cluster center c_i_ is represented by the matric *d*(*x_j_,c_i_*), number of clusters is represented by *C*, the number of data points in search space is denoted as *N* and *M *represents fitness degree where (m > 1).

Equation (1) can be solved by converting to an unconstraint problem by Lagrange multiplier. Membership degree and cluster centers are calculated using an alternate calculation cycle as they cannot be calculated simultaneously. Convergence is achieved by alternatively fixing the classes and calculating the membership function, followed by calculating the cluster centers by fixing the membership function. Algorithm 1 represents the pseudo code for FCM. 


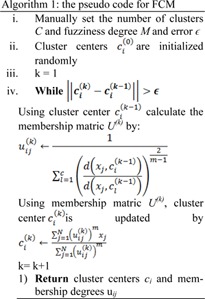


For image segmentation, the intensity value of the pixel *I *is represented by *x_i_*, total number of pixels is represented by* N*, the number of clusters (regions) is represented by *C, *distance between cluster center *c_j_* and pixel x_i_ is denoted by *d*^2^(*x_i_,c_j_*) and the membership degree between pixel *x_i_* and cluster *c_j_* is denoted by *u_ij_*.

### Cuckoo search based optimization

Inspired by reproduction strategies of cuckoo birds, cuckoo search optimization was proposed by (Yang and Deb, 2010[76]). Cuckoo lays egg on other bird's nest, based on this observation the following rules were proposed (Yang and Deb, 2010[76]):

1. Choosing a random nest, each bird lays one egg representing a set of solutions for the optimized problem.
2. With a fixed number of nests, there is a probability that the host might discover and discard the egg.
3. The nests containing the best solutions (egg) will be carried to the next iteration (new generation).

Levy flight (modeled after bird flight) is used in generating new solutions in cuckoo search, given by:


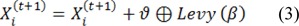


Where ϑ represents the step size and is determined by the scale of the problem (in this study set as 1), the product 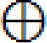 represents entry-wise multiplications. In an essence, random walkes are provided by Levy ﬂights with their random steps calculated from a Levy distribution for large steps having infinite mean and variance:


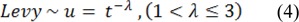


Essentially, the consecutive jumps and steps of a cuckoo search form a random walk process that obeys a heavy tail probability distribution. Algorithm 2 represents the pseudo code for performing the Cuckoo search (CS).


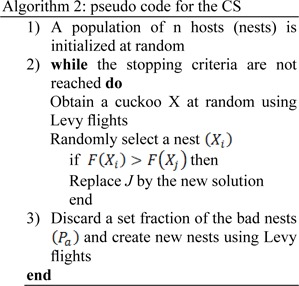


Stopping criterion can be based on the following conditions:

1. If after *n_erp_* iterations, no significant improvement is achieved on the objective function 

2. Number of the iterations reaching maximum *nb_maxiter_*

Based on recommendation by (Yang and Deb, 2010[76]) *P_a_* is set as 0.25 in this study. Cuckoo search is used for calculation of optimum cluster centers by minimizing the FCM objective function.

### Random walkers method

Graph-Cut (GC) based segmentation is an alternative to boundary based segmentation methods, being a semi-automatic segmentation the user is required to provide the seeds representing the background and the object to be segmented, GC represents the image pixels as nodes on a graph with weighted edges representing the adjacency between the pixels. By finding the minimum cost function between all possible cuts of the graph, the GC segments the image into background and the object (Boykov et al., 2001[14]). The main disadvantage of regular GC segmentation is the bad handling of weak edges and noisy images, to overcome this limitation many methods have been proposed to enhance the basic GC algorithm. One of such methods receiving a wide interest in medical imaging is the random walker algorithm (Andrews et al., 2010[5]; Grady, 2006[28]; Cui et al., 2013[20]; Grady and Sinop, 2008[29]). Random walkers segmentation was proposed by Grady (2006[28]), it is a supervised segmentation method meaning that a set of labels must be defined for each object prior to segmentation, this can be done interactively by the operator or be assigned automatically according to a predefined criterion. Random walker method segments the image by calculating the probability 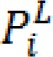 that a random walker starting at pixel '*i'* first reaching a pixel labeled *L*. 

The principle of random walker segmentation is the construction of an undirected graph *G= (V, E)* where the nodes *v* ∈ *V* correspond to image pixels and *e* ∈ *E *⊑* V *×* V* . Weight *W_ij_* is assigned to edge *e_ij_* connecting nodes *v_i_* and *v_j_* based on the following equation: 


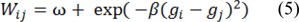


Where *g_i_* is the intensity at pixel *i* and *g_j_* is the intensity at pixel j. *β* is a scaling parameter set according to image contrast and ω is a regularization parameter that amounts to penalizing the gradient norm of *P^L^* (ω = 0 results in no regularization). 

Weight *W_ij_* can be described as the probability of the random walker crossing a particular edge, random walkers will cross edges more easily in case of more homogeneous edges created by a lower edge weight and thus region labels are decided more by the pixel distance to seeds labeled *L* and less by image features. Greater values of edge weight create less homogeneous edges thus making it harder for random walkers to cross edges and the region labels are decided more by the locations of strong edges. 

With the help of the circuit theory, Grady (2006[28]) showed that the connections between random walkers on a graph correspond to a combinatorial analog of the Dirichlet problem thus dramatically reducing calculation time by providing a convenient and simple method for the label probabilities computation. 

A Dirichlet problem can be defined as the problem of finding a harmonic function subject to certain boundary values. A Dirichlet integral could be represented as:


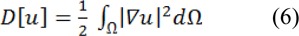


Where *u* represents a field and Ω represent the region (Grady, 2006[28]). The harmonic function minimizing the Dirichlet integral and satisfying the boundary condition can be achieved by the following Laplace equation:


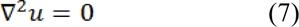


Let's denote *V_m_* as a set of seeded pixels and *V_u_* the set of non-seeded pixels, such that *V_u_* ∩ *V_m_* = ∅ and *V_u_* ∪ *V_m_* = *V*. It was shown that all of the probabilities 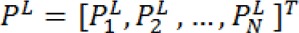 that each node (pixel) *V_i_* ∈ *V_u_* being assigned to label *L *can be obtained by minimization of:


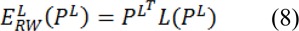


Where the probabilities of seeds *P^L ^*are assigned as: 


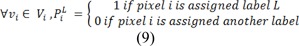


Where combinatorial Laplacian matrix of *L* is defined as:


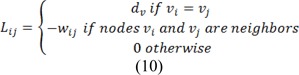


Where *d_v_* is the degree of the vertex of edge *v_i_* (sum of weights of all the edges *e_ij_* connecting *v_i_*), for 2D images the vertices will have degree of 4 or 8 and for 3D images the vertices could have a degree from 6 up to 26. Eq. 6 can be rewritten as:


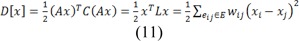


Where *C* is a diagonal matrix edge weights assigned to its diagonal and *A* is the incidence matrix defined as:


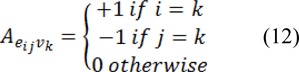


Eq. 11 can be decomposed as:


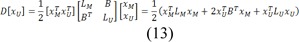


Where *x_U_* represents the probabilities of seeded and *x_M_* represents the probabilities of unseeded nods, critical points are determined by differentiating *D*[*x_U_*] with respect to *x_U_* as:


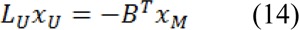


Which represents a system of linear equations where |*L_U_*| represents the unknowns. Using Eq. 9, the combinatorial Dirichlet solution can be found by solving the following equation for all labels:


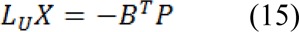


Only *L ˗ 1* systems must be solved as the sum of all probabilities at a node will be equal to zero:


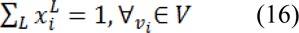


After minimizing 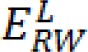 for each label *L*, the segmented region is obtained by calculating maximum probability of the label by:


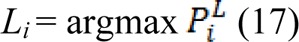


The workflow of the random walker method for image I can be summarized as:

1. Provide a set of marked pixels with L labels corresponding to desired segmentation regions

2. Map the image features such as intensities, texture information or other image features to edge weights and built the Laplacian matrix 

3. Perform the random walker and obtain segmentation label for each region.

In some segmentation tasks, the number of objects and their density estimation might be known prior to segmentation or can be generated from training and pre-labeling. In this study we utilize random walkers method with integrated priors (RW*_p_*) for enhancing the overall performance of regular random walkers method as discussed in (Grady and Sinop, 2008[29]).

**Figure 4:**
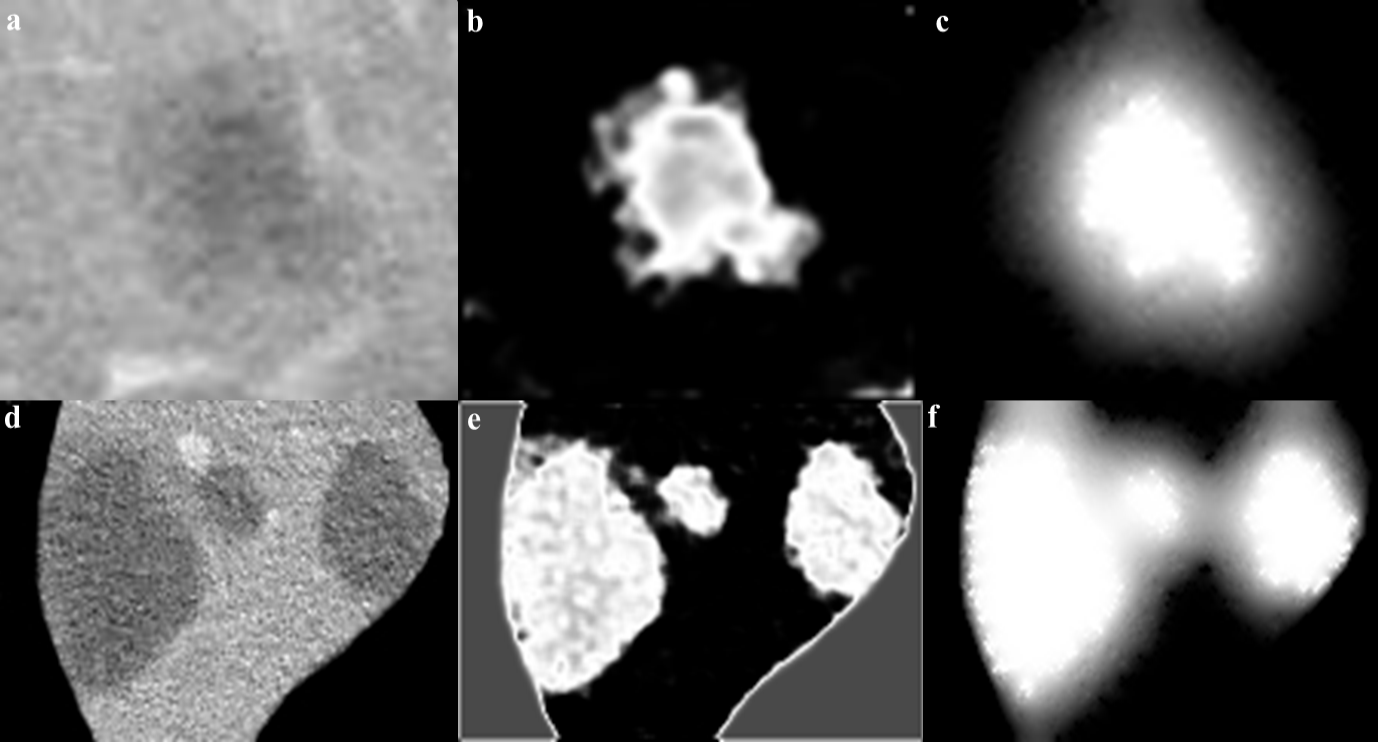
Cropped tumor image (a,d), CS-FCM clustering (b,e), corresponding probability (e,f)

**Figure 5:**
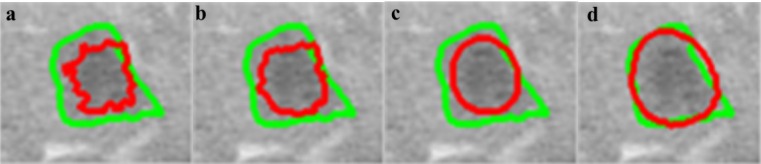
Effects of varying W_ij_ on segmentation (red line) compared to radiologist segmentation (green line), β=100 (a), β=10 (b), β=1 (c), β=0.1 (d)

**Figure 6:**
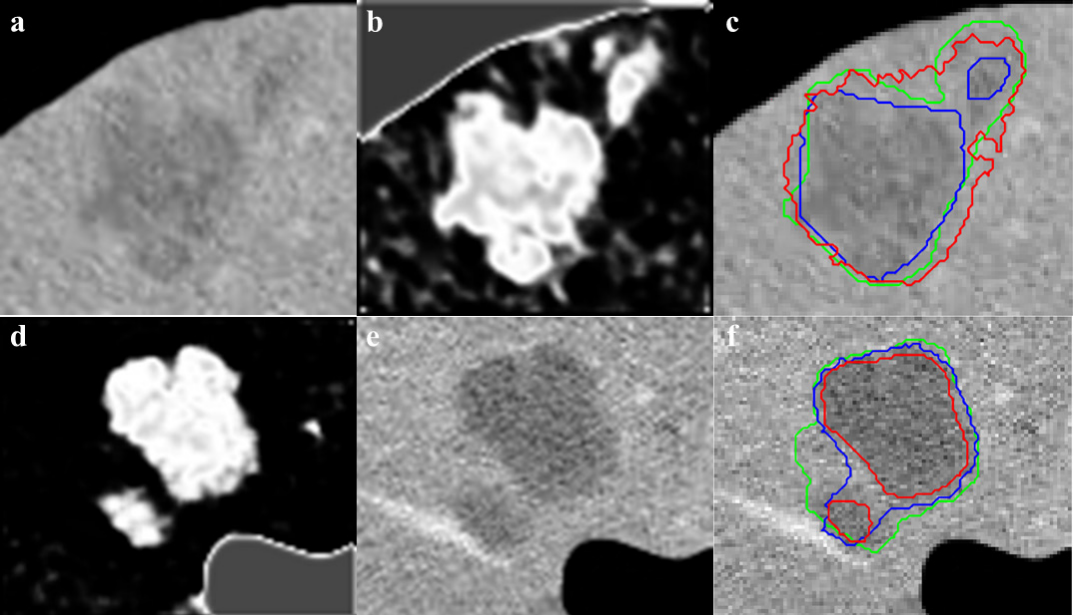
Cropped CT image with 22 mm and 7 mm tumors (a) and 32 and 9 mm tumors (d), initial CS-FCM clustering (b,e), radiologist segmentation (green outline) vs RW (red outline) (β=30) and RW_P_ (blue outline) (γ=0.003) (c,f)

The segmentation obtained by the CS-FCM is used for labeling of the pixels for the random walker segmentation. Figure 4(Fig. 4) shows the segmentation of tumors with the corresponding probability 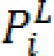, it should be noted that the values are pixel specific and are mapped to a gray scale and displayed for easy visualization and Figure 5(Fig. 5) illustrates effects of varying *W_ij_*. As segmentation of tumors is done inside the liver envelope, RW_P_ can be used to greatly enhance the segmentation results compared to regular RW algorithm. Figure 5(Fig. 5) illustrates a comparison between regular RW and RW_P_, as it can be seen, the use of RW_P_ greatly increases the accuracy of the segmentation. As evident from cropped CT images of tumors shown in Figure 6(Fig. 6), the liver image is quite noisy making accurate segmentation a challenging task. The main advantage of RW algorithm is the noise tolerance, as evident by many medical imaging tasks where RW algorithm has been shown to provide the most accurate segmentation (Bağci et al., 2011[8]; Chen et al., 2011[16]; Choubey and Agrawal, 2012[19]).

### Statistical performance measures

Before any discussion on the results a brief introduction of statistical performance measures utilized are given below, these statistics are calculated based on guidelines and the evaluation software by Taha and Hanbury (2015[70]). 

### Volumetric overlap error 

Volumetric overlap error (VOE) represents the number of pixels in the intersection of segmented region (A) and the ground truth (B), divided by the number of pixels in the union of A and B. A value of 0 % represents perfect segmentation while any increase in this value correlates to increased discrepancy between segmentation and ground truth. It can be calculated in percent from the following formula:


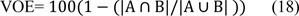


### Relative absolute volume difference

Relative absolute volume difference (RVD) expressed in percent, whereby the total volume of the segmented region is divided by the total volume of ground truth. It can be calculated by the following formula:


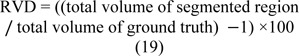


This measure should not be utilized solely to assess the performance of any segmentation method as a value of 0 (perfect segmentation) can also be obtained from an inaccurate segmentation, as long as the segmented region volume is equal to the volume of the ground truth. Please note that negative values represent under segmentation while positive values point to over segmentation.

### Dice similarity coefficient

Dice similarity coefficient (DSC) represents the overall performance of the image segmentation algorithm. It can be calculated by the following formula:


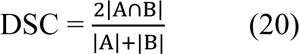


A value of 0 represents no overlap between the segmented region and ground truth while a value of 1 represents perfect segmentation.

## Results and Discussion

**Figure 7:**
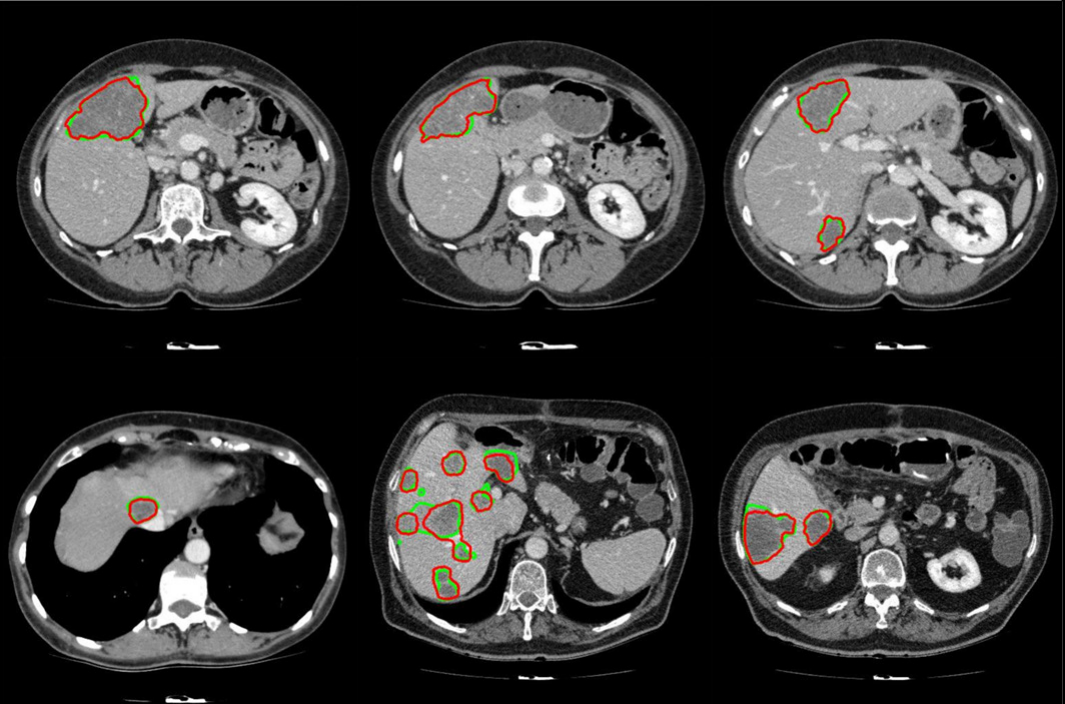
Examples of segmented liver tumors with the proposed framework, with green line representing ground truth and red representing the proposed segmentation

It should be noted that while all tumor sizes over 5 mm were included in 3Dircadb dataset segmentation, RECIST standard was the basis for the expert segmentation in MIDAS dataset. Tumors under 5 mm are not included in the segmentation as they are visible in only one or two slices in a CT series acquired with 2-3 mm slice thickness and usually offer no notable value for analysis. Apart from 3Dircadb dataset, almost all other publications are based on datasets with segmentation based on the RECIST standard. Figure 7(Fig. 7) illustrates a comparison of the radiologist segmentation and the segmentation from proposed method. The statistical performance of the proposed method compared to other methods from the literature is presented in Table 1(Tab. 1) (references from Table 1: Linguraru et al., 2012[47]; Vorontsov et al., 2014[71]; Masuda et al., 2011[49]; Shimizu et al., 2008[66]; Huang et al., 2013[39]; Stawiaski et al., 2008[68]; Cheng and Zhang, 2011[18]; Häme and Pollari, 2012[32]; Zhou et al., 2010[79]; Huang et al., 2014[40]). Unfortunately, although some researchers have utilized the 3Dircadb dataset in their liver segmentation methods, it seems that the 3Dircadb dataset has not been utilized for the development of tumor segmentation methods.

Based on the statistical performance, it can be assumed that the proposed method is amongst the most accurate segmentation methods proposed and tested for liver tumor segmentation from CTCE images, achieving a comparable or higher accuracy compared to other methods. In the case of 10 tumors from the MIDAS dataset, the proposed segmentation method was able to achieve an average DSC of 0.81 while the VOE was at 15.61 % and RVD was 4.02 %. Average DSC of 0.75, VOE of 22.78 % and RVD of 8.59 % was the performance of the proposed method on the 3Dircadb dataset containing 117 tumors, considering that the 3Dircadb dataset included many small tumors representing considerable difficulties in automatic segmentation, these results are promising. While preparing the LTSC '08 segmentation challenge (Deng and Du, 2008[22]), the organizers observed that VOE of 12.94 % and RVD of 9.64 % was the average performance of manual tumor segmentation by a human operator (with medical training) compared to ground truth segmentation by an expert radiologist. Although the relative volume difference (RVD) calculation and determining the liver tumor volume is of grave importance as discussed earlier, segmentation algorithms still can achieve a high score in this area while being quite inaccurate as the algorithm can still give accurate volume calculations while the segmented region is widely inaccurate compared to the ground truth. This can be also observed from the overlap error of different segmentation methods, as a method with high overlap error can have a low relative volume difference error.

The proposed segmentation method is comparable to other approaches developed with a runtime of around 30 seconds per slice, of which nearly 5 seconds were taken by random walker and the rest were used by CS-FCM for initial clustering. The proposed method is also viable as an alternative approach to manual segmentation by radiologists while being faster than manual segmentation for a CT volume containing many tumors with average manual segmentation time of 4.2 minutes per tumor (Häme and Pollari, 2012[32]). As discussed earlier, segmentation of liver tumors is a challenging task for automatic segmentation methods, based on these results it can be assumed that the proposed method is comparable to semi/interactive segmentation performance. 

Lower than expected performance of the segmentation methods for liver tumors can be associated with the vague boundaries of tumors making an accurate segmentation a challenging task. On the other hand, the small size of tumors results in increased statistical errors as a discrepancy of a dozen pixels can lead to a considerably increased error. Furthermore, from the dataset it is evident that many radiologists tend to over segment the tumor boundary and some even consider close tumors as a single tumor. However, there is no gold standard as the segmentation purely subjective and dependent on the radiologist, evident from MIDAS dataset where there is a disagreement of 9.8 % on the boundaries of ten tumors between five professional radiologists.

Due to the availability of a dataset (3Dircadb) containing radiologist segmentation of both liver and liver tumors, the tumor burden error (TBE) calculation (the difference between automatically and manually measured tumor burdens) of the proposed method has also been done. As discussed earlier, one of the most important variables for treatment monitoring and liver surgical planning is the tumor burden. Unfortunately, probably due to a lack of appropriate datasets, aside from (Linguraru et al., 2012[47]) no other researcher has reported the TBE for their method. The proposed method was able to achieve an average TBE of 0.84 % compared to TBE of 0.90 % reported by (Linguraru et al., 2012[47]), clearly collaborating with the results of the performance metrics in Table 1(Tab. 1).

**Figure 8:**
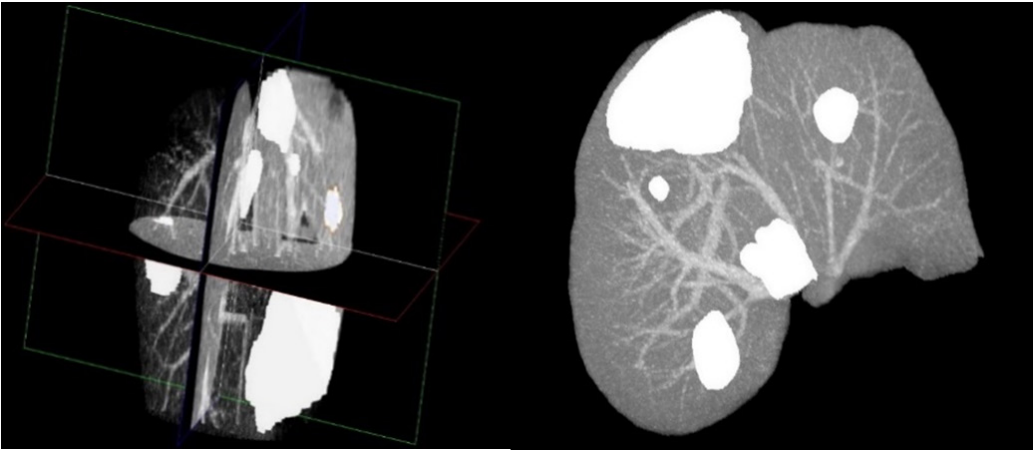
3D Reconstruction of a liver with segmented tumors by the proposed method with pathologies represented in white

After the extraction of liver and liver tumors from the CT series, 3D virtualization can be utilized to help the physician in better visualizing the liver and possible tumors inside the liver. This is done as going through a CT series on a slice by slice basis can be both tedious and time-consuming. Figure 8(Fig. 8) represents the 3D reconstruction of segmented tumors inside liver by the proposed method. As manual segmentations by radiologists tend to be subjective and to better evaluate the clinical value of the proposed segmentation, we utilized the expertise of a consultant radiologist with over 30 years teaching and research experience in medical imaging. In a blind evaluation and on a slice by slice basis, our expert compared the segmentation provided by the radiologist (considered as ground truth) in the dataset and the segmentation from the proposed method for tumors where the tumor size was more than 5 mm. Interestingly, our expert preferred the segmentation by the proposed method in 71.3 % of the tumor slices over the provided radiologist segmentation in the dataset, citing good tumor boundary tracking, speed and ease of utilizing the proposed method, further highlighting the need for an accurate and fast automatic tumor segmentation method for clinical applications. 

## Conclusions

Proper segmentation of liver and liver tumors is a prerequisite for any accurate CAD system utilized in liver cancer treatment planning and monitoring as accurate volume calculation and location estimation is the key in accurate prognosis. The proposed segmentation method was shown to provide accurate segmentation. One of the largest liver tumor datasets in the literature with varying contrast and enhancements were utilized for validating the results of the proposed method, the proposed method achieved excellent results in all instances with the average overlap error for tumor segmentation improved by almost 6 % compared to some of the best automatic methods in the literature. With a total runtime of about 16 minutes per patient, the proposed method is fast enough to be considered as a viable segmentation method for liver tumors. The proposed method was also able to achieve a tumor burden error of 0.84 %, well under the 10 % error threshold for clinical applications. The accuracy of the proposed method was further verified by a consultant radiologist for ensuring clinical applicability of the proposed method.

The proposed method is based on well-documented algorithms, making the implementation relatively easy for inclusion in any CAD system and can be easily expanded to other tumors and segmentation challenges from a medical perspective. Further development of the proposed framework can be automatic analysis and classification of the segmented tumors for an integrated liver CAD system for use in liver treatment planning as tumor grading is another important part of liver diagnosis and graphic processing unit based acceleration for decreased processing time.

## Conflict of interest

Authors declare that they have no conflict of interest.

## Figures and Tables

**Table 1:**
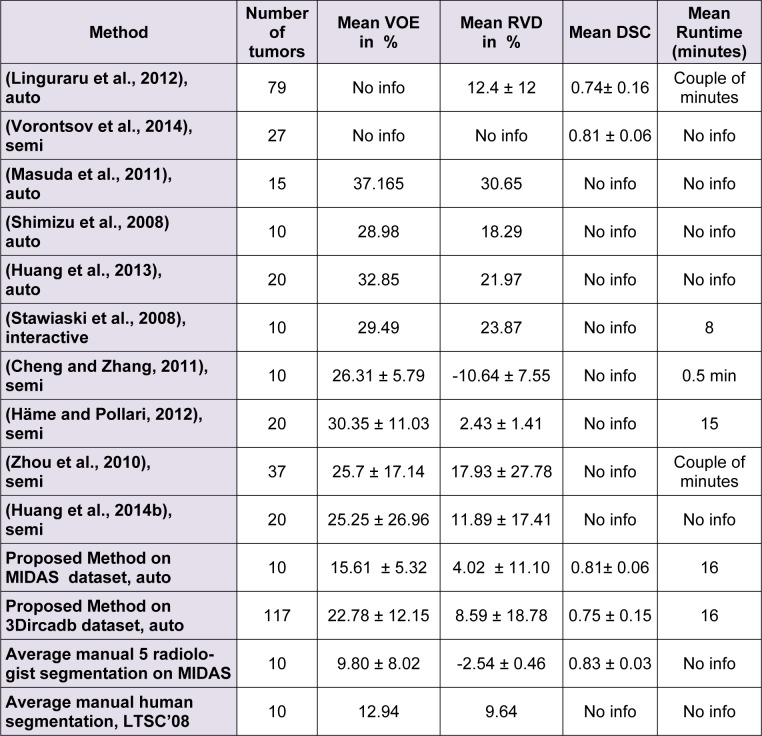
Statistical performance of the proposed method compared to some other proposed methods
